# Nepal Earthquake: An Experience from Ground Zero

**DOI:** 10.4269/ajtmh.18-0701

**Published:** 2019-04

**Authors:** Janak Koirala, Sangita Basnet

**Affiliations:** 1 Division of Infectious Diseases, Southern Illinois University School of Medicine, Springfield, Illinois;; 2 Pediatric Critical Care, Southern Illinois University School of Medicine, Springfield, Illinois

The Annual Conference of the Society of Internal Medicine of Nepal was in progress at Hotel Crowne Plaza Soaltee in Kathmandu, when an earthquake struck Nepal on April 25, 2015. It seemed as if the world was ending. The lights went out, and there was a state of panic in the room. People collapsed as they struggled to run toward the exit of the conference hall, appearing as though they were on a ship hit by a massive wave.

This massive tremor, a 7.8 on the Richter scale, the strongest since the great earthquake of 1934, destroyed a large part of central Nepal; ancient temples, palaces, and UNESCO World Heritage monuments were reduced to ruins. The quake was followed by numerous aftershocks that continued to rock the nation for more than a year. Thousands of people were buried under the rubbles of flattened homes in the capital city and surrounding hills. Millions more became homeless. Roads cracked open, sink holes big enough to engulf multistory buildings emerged, and giant rocks several feet high jutted out of the ground. Landslides caused roadblocks and avalanches created havoc, killing and injuring mountain climbers from around the globe.

On recovering from the initial shock to our cores, Nepali physicians who were present immediately jumped into action. We mobilized our organization, America Nepal Medical Foundation (ANMF), to perform surveillance and needs assessments on the ground in Nepal as well as gathering and dispatching help from the United States. The ANMF is a not-for-profit organization based in the United States with a sister chapter in Nepal consisting mainly of physicians. Its main mission is to uplift the health of the people of Nepal. As soon as the news of the earthquake broke on April 25, 2015, at around 1:00 am central time in North America, ANMF members in the United Sates started raising funds on social media. Nepalese and others who loved Nepal from around the world started giving. We quickly reached more than $1 million, even though the initial goal had just been $20,000. We set up an ANMF command center in one of the hospitals in Kathmandu and recruited more than 50 physicians who were eager to help. Many other volunteers from within and outside Nepal joined us.

The medical community in Kathmandu implemented their emergency workforce. Surgeons and nurses worked tirelessly around the clock without taking any breaks for days. Their compassion and generosity were evident in their attempts to selflessly care for the unfortunate. Some hospitals saw more than a 1000 trauma cases and performed 200–400 surgeries within the span of a few days. Many other hospitals were well equipped but could not function because of the damage caused by the mighty earthquake.

Our team visited the major teaching hospitals that had supply shortages within Kathmandu and Dhulikhel, a city 30 km east of Kathmandu. Every hospital we visited was over capacity. We had to walk around injured patients lying in the hallways, waiting rooms, and other empty spaces. Moreover, these hospitals were short on medical supplies, including intravenous fluids, antibiotics, and even supplies and equipment for trauma and orthopedic surgeries. We delivered boxes of these much-needed supplies to these facilities— supplies we acquired by knocking on supplier doors because all stores were closed after the earthquake.

Whereas the capital had suffered extensive damage, the earthquake’s epicenters were around Gorkha and Sindhupalchok, in the central region of Nepal. Our team drove in a pickup truck to Kavrepalanchok and Sindhupalchok, near one of the epicenters. Along the way, we saw destruction beyond our imagination. Rows upon rows of houses were destroyed and entire villages were flattened, leaving only rubbles of brick and wood visible on the ground. This was in stark contrast to the capital city, where most houses were still standing, except ancient temples, old palaces, and houses in certain pockets. Most modern buildings in Kathmandu are constructed with cement, concrete, and bricks and are supported by concrete pillars to withstand tremors. However, houses and huts in rural villages are built on mountain slopes and made of mud and bricks or stones. Some villagers lived in tents and homemade sheds. Others had nothing they could use for shelter and waited helplessly exposed to the elements for aid to arrive. Thankfully, the weather in Nepal during April–May is temperate, apart from wind gusts and cold nights. We distributed tents and blankets which we had brought with us on the truck.

When glancing from hilltops, the resplendent beauty of this tiny Himalayan nation was evident with its lush green hills, glorious shining mountain peaks, and plentiful waterfalls and rivers running through the valleys. However, when viewing the villages and towns on the ground, harsh reality struck a severe blow at the sight of unfathomable destruction, ruins, and loss of human lives and livestock. Even the mountain hillsides reminded us of the earthquake, as many hillsides were dotted with fresh landslides that continued to grow with each aftershock.

We were far from the only aid group; however, most were concentrated within the Kathmandu Valley as the extent of the damage prevented even an hour’s drive out of the valley. Our foundation mobilized more than 50 volunteer physicians from Nepal and United States providing medical and other relief services. We formed multiple teams, each consisting of medical and nonmedical volunteers, which included local youth volunteer groups. The Nepal Army provided transportation and security as we provided relief to more than 40 small towns and villages in the remote mountainous areas that are normally inaccessible by regular vehicles. A van carried our medical team and medical supplies, while a truck followed with daily villager needs such as food, blankets, and tents. As a preventive measure, our team also distributed water-purifying tablets, oral rehydration salts, hand soaps, and oral hygiene kits.

Our doctors performed examinations and provided health-care services under the open sky alongside local health-care workers. A baby was delivered inside a taxi by a local midwife in front of a damaged health center, lacking a safe, closed delivery room. Patients were moved to tents and temporary shelters, if available. All of this activity stopped the moment night fell because the world around us would be submerged in darkness due to the lack of electricity. Drinking water and toilet facilities were nonexistent in some communities. Despite the enormous amount of support pouring in from the world community, the rugged terrain made transportation a herculean task. This massive earthquake further complicated the problem by causing landslides and damages to the roads. Some of the hospitals and many health centers continued to provide services in tents as all new construction was put on hold by the government of Nepal for a year because of the concerns of more destruction by ongoing aftershocks.

Overall, more than 53,000 people received medical care from more than 2,600 health-care workers, and 8,856 people died because of the earthquake. Similar to any other natural disaster of this proportion, the relief and recovery efforts shifted from the initial stage of crisis for basic needs, including food, water, and shelter, to the next phase of rehabilitation and rebuilding. This second phase is still ongoing now, 4 years since the earthquake.

In the earthquake-affected areas, 462 health-care facilities were completely destroyed and an additional 765 sustained partial damage, making them unsafe for patient care. America Nepal Medical Foundation volunteers worked tirelessly to raise funds both online and through events in the United States. Our Nepal team received approval from the government of Nepal to rebuild 13 health posts with prefabricated materials. All 13 were built and handed over to the government within a year of the earthquake. When we went back to inspect these facilities, our team was greeted with marigold garlands and smiles by the villagers. The district health officer took our team to visit the Chautara district hospital, where we had built an outpatient clinic with an attached maternity ward and a delivery room. The physicians were seeing more than 100 patients per day in this small outpatient unit. We were appalled to see that inpatient and emergency care was still in tents more than 18 months after the quake. Two torrential monsoons, two hot summers, notwithstanding the bitter winters—it was unimaginable. It was a hot day; there were flies buzzing around and a loud fan in the tattered and patched tent which was the emergency ward. There was a 2-year-old boy with high fever and seizures, most likely suffering from febrile seizures or even something more serious. The heat was not helping. We discovered that there would be a long delay before anything would be built there. Therefore, our ANMF team in the United States raised more than $80,000 and provided assistance to construct a beautiful building consisting of a medical ward and an emergency room.

Currently, Nepal has an adequate number of basic health man power produced by 20 medical schools. However, there is a lack of much-needed specialists in trauma, infectious diseases, critical care, and psychological and physical rehabilitation. Developing more expertise in these disciplines and establishing more trauma centers and rehabilitation services would make a positive impact in the future. A massive public health campaign is another absolute requisite for the prevention of infectious disease outbreaks. Similar to other major natural calamities in resource-poor nations, outbreaks of diarrheal diseases and other food- and waterborne diseases are imminent among people living in shelters because of the lack of access to toilet facilities and clean potable water. This is a public health emergency needing urgent intervention in its immediate post-disaster period. However, learning from the Haiti experience and other disasters, the Nepal government, WHO, and international communities brought awareness to the people about water, sanitation, and hygiene and took preventive strategies such as distribution of hygiene kits and rebuilding toilets. Our team also visited rural villages with public health students and distributed hygiene kits and educated the villagers and children about proper hygiene and sanitation. There were no major outbreaks in post-earthquake Nepal. Based on our data from one medical center, most of the post-earthquake–related infections were wound infections acquired in the hospitals. Although there was a small outbreak of typhoid, it was contained quickly by taking appropriate preventive measures.

The swift and effective reaction to the 2015 Nepal earthquake is an example of what can be achieved when the world comes together to aid during a massive natural disaster. Both the people of Nepal and the international community jumped into action and worked together side by side, complementing each other’s operations of rescue, relief, rebuild, and rehab. Nepal is still going through a long recovery process. Many of the houses have been rebuilt, but many other villagers are still living in temporary homes. When we visited 3 years later, we could still see tiny homes with shiny metal roofs scattered over the hillsides. These homes are built with corrugated sheets, bamboo, and prefabricated materials. Many villagers are still displaced and living somewhere else. Health posts and hospitals are still using the prefabricated buildings, as the original facilities are still closed and yet to be reconstructed. Although the overall recovery process has progressed well, there is a long way to go. However, the thousands of lost lives will never be replaced.

